# Revisiting the Hispanic Paradox: Neighborhood Characteristics and Life Satisfaction for Older Immigrants

**DOI:** 10.1093/geroni/igaf122.3492

**Published:** 2025-12-31

**Authors:** Morgan Stangl, Lauren Gil Hayes, Yi Wang, Man Guo

**Affiliations:** University of Iowa, Iowa City, Iowa, United States; University of Iowa, Iowa City, Iowa, United States; University of Iowa School of Social Work, Iowa City, Iowa, United States; University of Iowa, Iowa City, Iowa, United States

## Abstract

Nativity status (foreign-born, FB vs. native-born, NB) is a social determinant of well-being, yet its impact on life satisfaction may vary depending on neighborhood characteristics. Current literature discusses the Hispanic Paradox, where FB Hispanics report higher life satisfaction despite immigration challenges, attributed to strong family support. However, neighborhood characteristics remains underexamined, offering new insights. The current study seeks to explore 1) Whether the association between neighborhood characteristics (cohesion/disorder) and life satisfaction is stronger for FB middle-aged/older adults than for NB middle-aged/older adults and 2) if this association varies by race (White, Black, Hispanic). This study utilizes data from the 2018 Health and Retirement Study (HRS) Psychosocial Leave-Behind Questionnaire, a nationally representative survey of middle-aged/older adults, including both NB (N = 4,398) and FB (N = 722). Life satisfaction was regressed on neighborhood cohesion and disorder separately, controlling for demographics and socioeconomic factors. Interaction terms between neighborhood characteristics and nativity status were added to test moderation. These interaction models were then stratified by race/ethnicity. Results show that FB middle-aged/older adults are more sensitive to neighborhood characteristics, with a significant interaction between nativity and neighborhood disorder (β = 0.15, p < 0.001). Neighborhood cohesion shows a strong positive association with life satisfaction for FB Hispanics (β = 0.38, p < 0.001). Neighborhood disorder has a strong negative association for FB Hispanics (β = -0.32, p < 0.001). These findings suggest that neighborhood characteristics may play a role in shaping life satisfaction, particularly for FB middle-aged/older Hispanic adults. These findings provide another dimension in understanding the Hispanic Paradox.

